# One health approach to chagas disease: a systematic review on the integration of human, animal, and environmental health

**DOI:** 10.3389/frhs.2026.1818840

**Published:** 2026-06-16

**Authors:** Kaic Santos Silva Pereira, Ricardo Evangelista Fraga, Eliana Amorim de Souza, Márcio Borba da Silva, Edma Santos Antonio, Alexandre Schiavetti

**Affiliations:** 1 Universidade Estadual de Santa Cruz, Ilhéus, Bahia, Brazil; 2 Instituto Multidisciplinar em Saúde, Universidade Federal da Bahia, Vitória da Conquista, Bahia, Brazil

**Keywords:** American trypanosomiasis, neglected tropical diseases, public health, sustainable development goals, *Trypanosoma cruzi*

## Abstract

**Introduction:**

Chagas disease ranks among the most important Neglected Tropical Diseases in the Americas. Given its epidemiological, social, and ecological complexity, the One Health approach has been increasingly recognized as a strategic framework for its integrated management. The aim of this research is to investigate how the One Health approach has been applied in Chagas disease studies.

**Methods:**

This study consists of a systematic review (2008–2024), with searches conducted in the Web of Science®, PubMed®, Scopus®, and Dimensions® databases, using descriptors combined with Boolean operators. The analysis included (i) scientometric methods supported by VOSviewer® software, (ii) systematization of the collected data in structured spreadsheets, and (iii) graphical visualization through a chord diagram (Flourish®).

**Results:**

The most prominent researchers were Ricardo Castillo-Neyra and Michael Levy. A strong contribution from the Americas was observed in studies addressing Chagas disease and the One Health approach. Of the 227 documents retrieved, 68 met the inclusion criteria. A predominance of studies focused on animal health (41.2%) was identified. Only 11.8% of the studies integrated the three core components of One Health (human, animal, and environmental). Although topics such as surveillance, vectors, and public health are highlighted, the data reveal that actions largely remain sectoral and fragmented.

**Conclusions:**

This systematic review highlights that although progress has been made in applying the One Health perspective to Chagas disease, it is still necessary to strengthen the effective integration among the three components of the approach.

**Systematic Review Registration:**

doi.org/10.17605/OSF.IO/5HVA8

## 1 Introduction

Chagas disease, or American trypanosomiasis, is among the Neglected Tropical Diseases (NTDs) with the greatest impact in the Americas. It is estimated that more than 7 million people are infected worldwide, with more than 1.5 million presenting severe chronic clinical forms, particularly Chagas cardiomyopathy (1, 2). The disease is caused by the protozoan *Trypanosoma cruzi* and is primarily transmitted by insect vectors of the subfamily Triatominae (kissing bugs) (3). In addition to vector-borne transmission, other relevant routes include vertical, transfusional, oral, accidental, and organ transplantation-related transmission (4), underscoring the complexity of its epidemiological dynamics.

Beyond its parasitic etiology, Chagas disease expresses a deeply rooted sociospatial dimension shaped by structural inequalities. Its persistence as a public health problem is closely associated with contexts of poverty, including precarious housing, inadequate sanitation, fragile health systems, and environmental degradation (5). These determinants highlight the limitations of reductionist biomedical approaches and underscore the need for interdisciplinary frameworks capable of addressing the broader ecological, social, and territorial determinants of transmission.

In parallel, Chagas disease imposes a continuous and often underestimated burden on health systems in low- and middle-income countries. Because most morbidity manifests years after the initial infection, patients typically seek health services only after developing chronic cardiomyopathy and/or digestive complications, conditions that require long-term follow-up, repeated diagnostic testing, hospitalizations, and, in severe cases, complex and costly interventions. These demands directly compete with other priority conditions in already resource-constrained systems. The World Health Organization emphasizes that the medical costs associated with the chronic clinical forms of Chagas disease may substantially exceed investments in preventive vector control strategies, underscoring the critical role of well-trained primary healthcare teams in improving case detection, etiological treatment, and long-term follow-up. This reality places additional pressure on workforce capacity and continuity of care within health systems (1, 6).

Economic modeling studies indicate that Chagas disease generates substantial annual costs for health systems, in addition to a high burden of disability, measured in Disability-Adjusted Life Years (DALYs). These impacts encompass not only direct medical expenditures but also indirect losses resulting from reduced productivity, work absenteeism, and broader social consequences. Such effects tend to be more pronounced in developing countries, where delayed diagnosis and inequalities in access to etiological treatment often lead to the management of complications at advanced stages, substantially increasing costs. Recent reviews on the economic burden of the disease reinforce this scenario, reporting significant annual per-patient expenditures and highlighting that the cumulative economic burden poses a structural challenge to the financing and operational capacity of health systems in endemic areas (6–8).

In Brazil, the annual economic burden of the chronic form of Chagas disease is estimated to reach US$ 11.44 billion, corresponding to approximately 0.23% of the Gross Domestic Product (GDP), with an average lifetime cost of US$ 45,034 per affected individual. Direct medical expenditures account for approximately 72% of the total lifetime economic burden, while indirect costs represent about 28%. In addition, the annual direct medical costs associated with the disease consume around 11% of the budget of the Ministry of Health, highlighting the substantial financial impact of Chagas disease and its relevance as a structural constraint on the sustainability of the public health system (9).

In this context, the One Health approach emerges as a promising framework for addressing complex and multifactorial conditions such as Chagas disease. By integrating the domains of human, animal, and environmental health, One Health recognizes their interdependence and promotes coordinated, intersectoral responses to health threats (10–12). This perspective becomes even more relevant when aligned with the Sustainable Development Goals (SDGs) of the 2030 Agenda [particularly SDG 3 (Good Health and Well-Being) and SDG 15 (Life on Land)] which emphasize the urgency of integrated strategies capable of simultaneously promoting health equity and environmental sustainability (13, 14).

The ecological complexity of Chagas disease, characterized by the presence of multiple vectors, diverse animal reservoirs, and heterogeneous ecological contexts, provides fertile ground for the operationalization of the One Health framework. Processes such as land-use and land-cover change, deforestation, habitat fragmentation, and climate change have directly influenced vector distribution and the emergence of new transmission foci (15). These transformations call for analytical and operational approaches that transcend disciplinary silos and integrate health, environment, and territory.

Against this backdrop, this study aims to examine how the One Health approach has been applied in scientific studies on Chagas disease through a systematic review, complemented by a bibliometric (scientometric) analysis. Particular attention is given to strategies that integrate the human, animal, and environmental health components. By critically synthesizing the available literature, this review seeks to contribute to the strengthening of more integrated, sustainable, and socially and ecologically sensitive responses to this historically neglected disease.

## 2 Materials and methods

This study was conducted following the guidelines of the “Preferred Reporting Items for Systematic Reviews and Meta-Analyses” (PRISMA) 2020 (16). The review protocol was developed with a focus on the interdependence among human, animal, and environmental health dimensions and was registered on the Open Science Framework (OSF) (DOI: 10.17605/OSF.IO/5HVA8).

### 2.1 Search strategy

The search was conducted in four databases: Web of Science®, PubMed®, Dimensions®, and Scopus® (searches were performed on July 30, 2025). To ensure sensitivity and specificity in the search strategy, controlled descriptors were used, extracted from the MeSH (Medical Subject Headings) and DeCS (*Descritores em Ciências da Saúde*—Health Sciences Descriptors) vocabularies. The terms employed included: “Chagas Disease” [MeSH/DeCS], “Trypanosoma cruzi” [MeSH/DeCS], “One Health” [MeSH], and “Environment and Public Health” [DeCS]. The descriptors were combined using Boolean operators, adapted to the specific requirements of each database (Table 1).

**Table 1:** Search strategy into electronic databases for systematic review.

Database	Advanced search	Nr of records
PubMed®	(“One Health”) AND (“Chagas Disease” OR “Trypanosoma cruzi” OR “American trypanosomiasis”)	67
Dimensions®	(“One Health”) AND (“Chagas Disease” OR “Trypanosoma cruzi” OR “American trypanosomiasis”)	36
Web of Science®	(“Chagas Disease” OR “Trypanosoma cruzi”) AND (“One Health”)	62
Scopus®	TITLE-ABS-KEY(“One Health”) AND TITLE-ABS-KEY(“Chagas Disease” OR “Trypanosoma cruzi” OR “American trypanosomiasis”)	62

### 2.2 Eligibility criteria and study selection

The study included scientific papers with full text available, published from January 2008 to December 2024, describing original empirical research (quantitative, qualitative, or mixed methods) that addressed Chagas disease and One Health. Excluded from this review were all publications that had not undergone peer review, literature reviews, editorials, conference proceedings, technical reports, theses, dissertations, and other documents classified as gray literature. This selection aimed to ensure methodological consistency, scientific validity, and comparability among the included studies.

The screening process was carried out in two sequential stages. In the first stage, titles and abstracts were assessed based on the eligibility criteria. In the second stage, the full texts of potentially eligible studies were evaluated for thematic relevance and methodological rigor. Discrepancies in selection were resolved by consensus between two reviewers and, when necessary, with the assistance of a third evaluator.

### 2.3 Data extraction and analysis

The data analysis for this study was conducted in two complementary stages: (i) scientometric analysis and (ii) systematic review with content analysis.

VOSviewer® was employed to perform the scientometric analysis. The PubMed® database was selected for this analysis as it yielded the largest number of retrieved documents, albeit only marginally higher than Web of Science and Scopus. Using VOSviewer®, co-occurrence networks of terms and co-authorship relationships were generated. This approach allowed for the identification of predominant thematic clusters, central keywords, established conceptual areas, and underexplored gaps. The analysis of clusters and term centrality enabled an integrated understanding of the emerging conceptual and methodological dimensions in the literature.

For the systematic review, the data were analyzed descriptively and interpretatively, combining scientometric and content analysis techniques. Data extracted from the selected studies were organized in a structured spreadsheet containing the following variables: author(s), year of publication, country of origin, study type and objectives, methodological approach, components of the One Health triad (human, animal, and environmental), and main findings (Supplementary Table S1). Data extraction was performed by one reviewer and subsequently checked by a second reviewer to ensure accuracy and consistency. Due to the substantial methodological heterogeneity of the included studies, no formal risk of bias assessment was performed.

Based on a full-text review of the selected papers, it was possible to qualitatively and quantitatively categorize the content of the studies, understanding how interactions among the human, animal, and environmental health domains are reflected in the discourse and methodologies. The final synthesis was structured according to a qualitative categorization framework, as described in Table 2, which systematizes the main components of analysis without representing a quantitative frequency assessment. This framework was developed inductively by the authors based on the thematic content of the included studies.

**Table 2:** Topics observed in studies for each component of the One health approach in chagas disease.

Component	Topic	Description*
Human Health	1	Collection and analysis of human biological samples
	2	Epidemiological studies (prevalence, incidence, spatial distribution)
	3	Socioeconomic aspects (housing, income, education, sanitation, access to healthcare)
	4	Population mobility
	5	Access to diagnosis and treatment
	6	Educational actions (prevention, awareness, social participation)
	7	Risk perception and knowledge about the disease
	8	Family or community disease history
	9	Economic and financial assessment of the disease
	10	Use of insecticides as a vector control measure with human impact
Animal Health	1	Entomological surveys and laboratory analyses of vectors
	2	Presence of domestic or wild animals in household environments
	3	Biological tests in animals to detect infection
	4	Identification and distribution of reservoir species
	5	Management practices (rearing, feeding, shelter)
	6	Human–animal interactions
	7	Use of insecticides in vector control
Environmental Health	1	Georeferencing and mapping of risk areas
	2	Analysis of land use and cover (deforestation, urbanization)
	3	Physical conditions of housing and surroundings (debris, roofs, waste, sanitation)
	4	Climatic and seasonal variables (temperature, rainfall, humidity)
	5	Anthropogenic activities (agriculture, extractivism, mining)
	6	Lack or inadequacy of sanitation infrastructure
	7	Vegetation type and proximity to wild areas
	8	Occupation of environmentally risky areas (riverbanks, slopes, reserves)
	9	Impact of insecticide use on the environment

* Source: The authors.

Studies were assigned to one or more components of the One Health approach (human, animal, and environmental) based on the presence of at least one topic corresponding to each component (Table 2). When studies addressed topics spanning multiple components, they were classified into all relevant categories (e.g., studies addressing both animal reservoirs and human infections were categorized as “animal and human health”). No hierarchical prioritization was applied, and the categories were not mutually exclusive. Accordingly, seven possible classification categories were defined: (1) human health; (2) animal health; (3) environmental health; (4) animal and human health; (5) environmental and human health; (6) environmental and animal health; and (7) animal, human, and environmental health. The assignment to categories was not based solely on the presence of isolated terms or topics, but required evidence that the dimension was analytically incorporated into the study, whether through data, indicators, or interpretative discussion. An illustrative example of the categorization process is provided in Supplementary Box S1.

A chord diagram (Figure 3) was developed to represent the interrelationships among the components of the One Health approach (human, animal, and environmental health) in studies on Chagas disease. The data were organized into a co-occurrence matrix reflecting the frequency of associations among these components and their combinations. The matrix was exported in CSV format and uploaded to the Flourish platform, where nodes (representing the analytical categories) and connections (indicating the relationships among them) were configured. The thickness of the chords represents the strength of these associations, enabling the identification of patterns of integration and highlighting both predominant connections and gaps among the components.

## 3 Results

**Figure 1:**
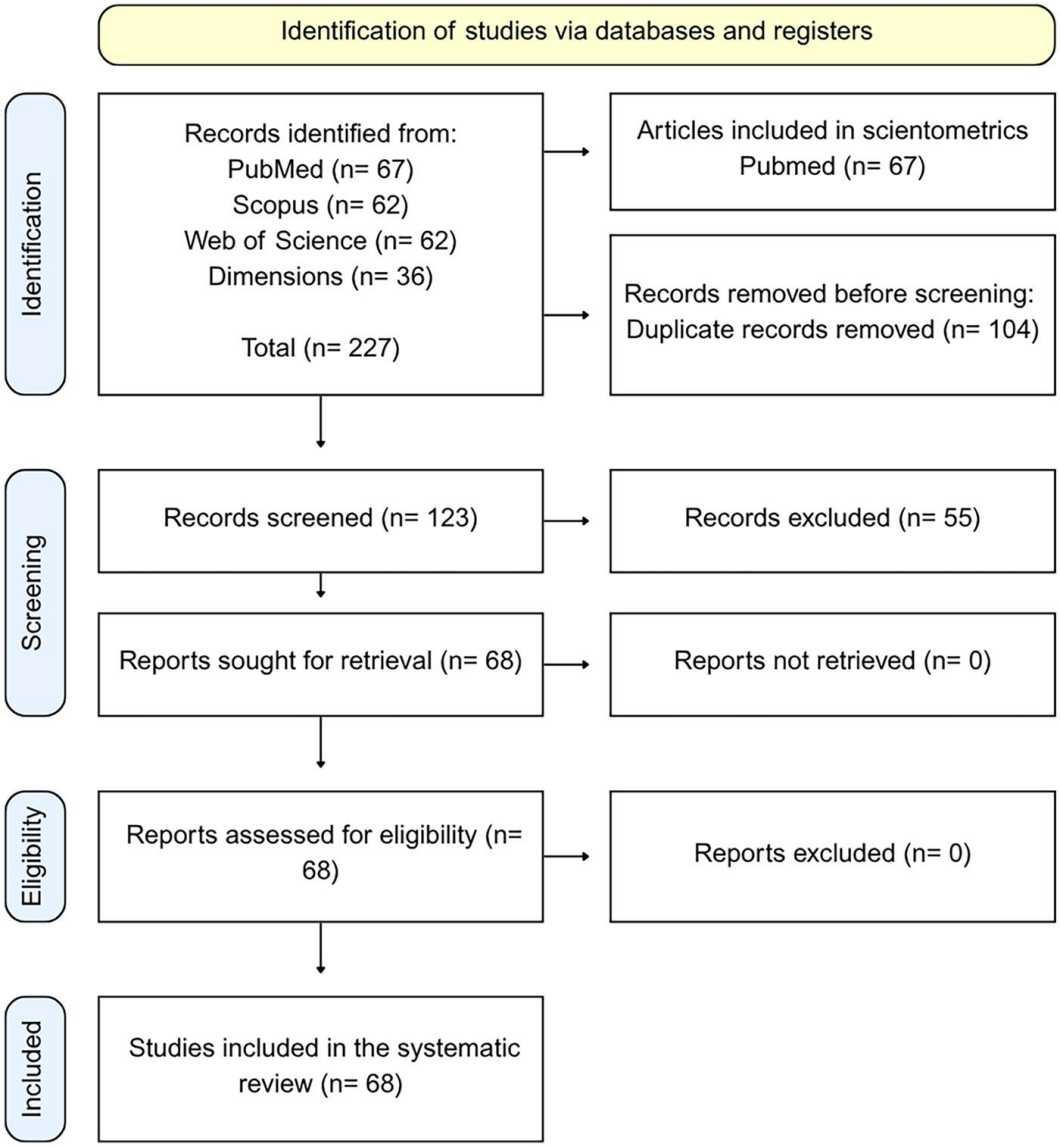
PRISMA 2020 flow diagram of study selection.

**Figure 2:**
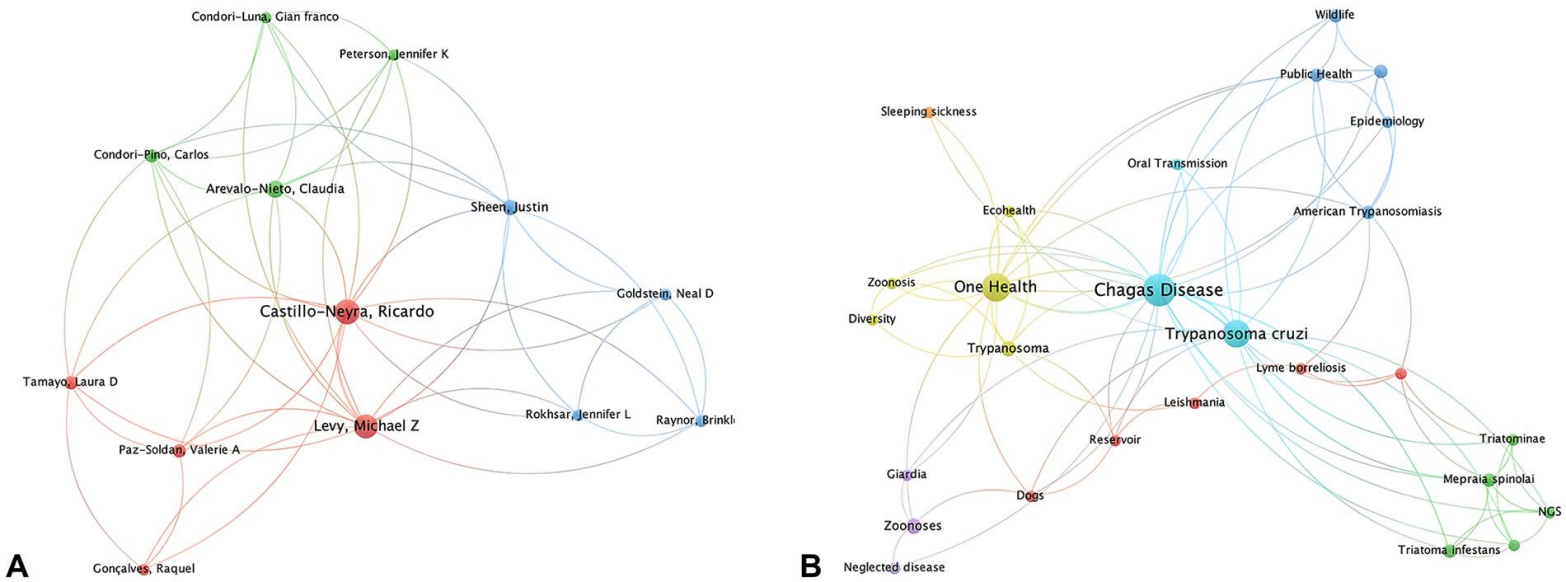
Scientometric networks of scientific production on chagas disease and One health: **(A)** authors and **(B)** keywords and thematic clusters.

The scientometric analysis was conducted using 67 documents retrieved from the PubMed® database (Figure 1). This analysis identified the most influential authors, the main recurring terms in the studies examined, and patterns of institutional collaboration (Figure 2).

The co-authorship network identified Ricardo Castillo-Neyra (USA and Peru) and Michael Z. Levy (USA and Peru) as the most prominent authors in terms of scientific output, with 12 and 11 publications respectively, and collaboration, both with a total link strength of 29. Claudia Arevalo-Nieto (Peru) also stands out with 5 publications and a link strength of 17, highlighting her participation in consistent collaborative networks. Raúl Araya-Donoso (USA) and Antonella Bacigalupo (UK) each have 3 publications and a link strength of 15, establishing them as authors of intermediate relevance in the field of Chagas disease research from a One Health perspective (Figure 2A).

The descriptors revealed in the scientometric analysis (Figure 2B) highlight the central themes of the publications. The term “Chagas disease” emerged as the most frequent (*n* = 22) and exhibited the highest total link strength (strength=40). “One Health” was the second most prominent (*n* = 16; strength=31), reflecting the increasing incorporation of this interdisciplinary approach in the context of Chagas disease. The etiological agent “*Trypanosoma cruzi*” was also highly cited (*n* = 15; strength=30). More specific terms, such as “*Mepraia spinolai*” (*n* = 3; strength=10) and “American trypanosomiasis” (*n* = 3; strength=9), were less frequent but indicate a deeper focus on entomological and terminological aspects that complement the field of study.

Scientific production on Chagas disease in association with the One Health approach is concentrated in a diverse set of institutions across different countries, reflecting the interdisciplinary and international nature of the topic. Among them, Brazilian organizations stand out, such as the Fundação Oswaldo Cruz (Instituto René Rachou) (*n* = 2; strength = 2) and the Universidade Estadual de Montes Claros (*n* = 2; strength = 2). In the Latin American context, the Universidad Peruana Cayetano Heredia is notable with two units involved: the One Health Unit in Lima (*n* = 3; strength = 0) and the Zoonotic Disease Research Laboratory in Arequipa (*n* = 2; strength = 2), demonstrating the country's regional leadership at the interface of zoonoses and public health. The Universidad San Sebastián (Chile), Arizona State University (USA), and the University of Pennsylvania (USA) also contribute relevant publications, albeit with lower connectivity (*n* = 2; strength = 1 or 2). This panorama highlights an expanding scientific network, characterized by strong collaborative hubs in Brazil and Peru, with increasing participation of North American and Chilean universities in the discussion of One Health and Chagas disease.

A total of 227 papers were retrieved from the four databases used (Figure 1). After removing duplicates and applying the inclusion and exclusion criteria, 68 studies were selected for full-text review (Supplementary Table S1).

The temporal evolution of scientific production shows a marked increase in recent decades. The year 2024 accounted for the highest number of publications (*n* = 12; 17.6%), followed by 2023 (*n* = 11; 16.2%). Between 2020 and 2022, output remained stable, with 6–7 publications per year (8.8%–10.3%). In earlier years, production was more limited, with 4–5 papers annually between 2016 and 2019 (5.9%–7.4%). Prior to 2015, records were sporadic, with only one article published in 2008, 2011, 2012, and 2014 (1.5% each), highlighting the recent consolidation of the One Health approach in Chagas disease research.

The geographical distribution of the 68 studies reveals a wide diversity of contexts. Brazil was the most representative country (*n* = 16; 23.5%), followed by Mexico (*n* = 7; 10.3%), the United States (*n* = 6; 8.8%), and Peru (*n* = 6; 8.8%). South America, as a region, was the focus of 10 studies (14.7%), while Argentina and Bolivia were individually mentioned in 3 papers each (4.4%). Other countries with lower frequency (*n* = 2; 2.9%) include Chile, Colombia, Costa Rica, and Guatemala. Countries with only one occurrence (*n* = 1; 1.5%) include Belize, Ecuador, Honduras, Nicaragua, Kenya, Switzerland, the Caribbean, Venezuela, and generic mentions of Europe, reflecting occasional yet relevant contributions.

A wide range of methodologies was employed in the studies reviewed, with a strong prevalence of laboratory and diagnostic approaches. Molecular techniques were the most frequently used (21/68; 30.9%), followed by serology (10/68; 14.7%) and interviews or participatory studies (10/68). Computational modeling appeared in 9/68 papers (13.2%), indicating the increasing use of predictive tools. Entomological surveys (6/68; 8.8%) and geoprocessing (4/68; 5.9%) were also recurrent. Less common methodologies included ecological analysis (3/68; 4.4%); use of secondary data (3/68); phylogenetic analysis, controlled experiments, genotyping, clinical case reports, and environmental monitoring (1/68; 1.5% each).

**Figure 3:**
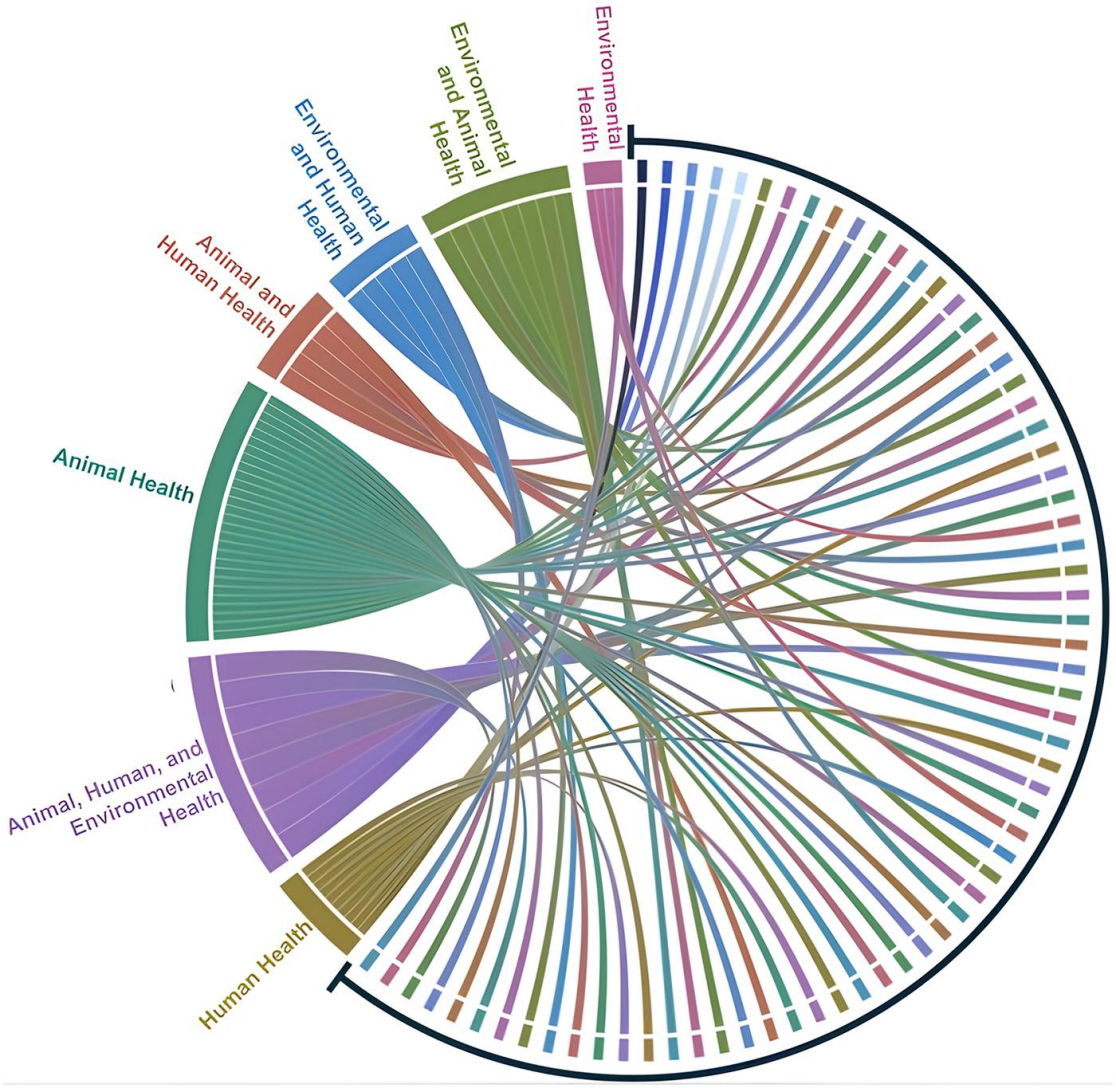
Chord diagram illustrating the integration of the three components of the One health approach (human, animal, and environmental health) in studies on chagas disease. Tool used: Flourish®.

Regarding the three components of the One Health approach, studies predominantly focused on animal health (*n* = 28/68; 41.2%). Only 8/68 papers (11.8%) articulated an integrated perspective encompassing the three components (human, animal, and environmental), indicating the still limited presence of truly interdisciplinary analyses. Another 8/68 studies simultaneously addressed the animal and environmental components; 5/68 (7.4%) examined the environmental and human components; and 5/68 (7.4%) integrated animal and human health. Human health was explored in isolation in 10/68 papers (14.7%), and environmental health in 4/68 (5.9%) (Figure 3).

## 4 Discussion

The first study retrieved in the present review linking Chagas disease to the One Health approach was published in 2008. This finding aligns with the historical development of the One Health concept. The recognition that human health is interconnected with animal and environmental health dates back to the 1800s. However, the term One Health was formally consolidated as an integrated approach to addressing infectious diseases in 2008, following the publication of the final report of the One Health Initiative Task Force, entitled “One Health: A New Professional Imperative” (17).

Since 2008, a substantial increase in scientific production associating One Health approach with Chagas disease has been observed. This expansion has become particularly pronounced over the past five years, signaling the consolidation of an emerging field and its increasing relevance in the face of contemporary public health challenges. Recent global crises, such as the COVID-19 pandemic, have reinforced the urgency of integrated strategies to address infectious diseases, highlighting the transformative potential of interdisciplinary and intersectoral perspectives inherent to One Health (18). This movement also resonates with historical experiences, such as the international mobilization during the H5N1 avian influenza outbreak in Hong Kong in 1997, when pandemic risk prompted strengthened interinstitutional dialogue and contributed to the consolidation of the concept (19).

The co-authorship network revealed the presence of well-established international collaborations. The collaboration between researchers from developed countries and Latin American scientists constitutes a dynamic hub of scientific production, encompassing areas such as epidemiological surveillance, molecular biology, vector ecology, and spatial modeling (20, 21). Studies such as *Continuing evidence of Chagas disease along the Texas–Mexico border* (22) illustrate the potential of scientific cooperation to integrate serological, vector-based, and epidemiological analyses into more holistic approaches capable of robustly addressing the complexity of Chagas disease transmission.

The scientometric analysis of keyword co-occurrence indicates a progressive expansion of the thematic scope of research. However, One Health remains peripheral, suggesting limited integration across domains. The recurrent presence of descriptors associated with vectors reinforces the central role of entomology in sustaining transmission. The emergence of the term *dogs* is noteworthy, highlighting the role of dogs as epidemiological sentinels in several studies, given their susceptibility and integration into the transmission cycle (23–25). The retrieved keywords indicate an emerging trend toward integrating biomedical, ecological, and social dimensions.

At the geographical level, Brazil stands out as the country with the highest volume of publications on the topic, reflecting both its scientific relevance and the epidemiological magnitude of the disease. The II Brazilian Consensus on Chagas Disease estimates that between 1.9 and 4.6 million people are infected in the country (26), underscoring the urgency of integrated strategies for surveillance, prevention, and control. Studies such as that by Campos et al. (27), which analyzed the occurrence, natural infection, and spatial distribution of triatomines in Montes Claros, Minas Gerais, demonstrate the importance of territorial approaches that go beyond surveillance of the acute phase, incorporating the follow-up of chronic cases and the continuous monitoring of vector foci (28). These findings reinforce the strategic role of territory and primary health care as operational pillars for the effective implementation of the One Health approach.

At the continental scale, Latin America accounts for the largest share of scientific production on Chagas disease, reflecting its historical status as an endemic region (29). Estimates from the Pan American Health Organization indicate that between 7 and 7.5 million people are infected worldwide (30). However, in Latin America alone this number may reach approximately 8 million individuals, of whom about 70% are unaware of their serological status (31). This scenario highlights persistent gaps in case detection, follow-up, and access to etiological treatment, reinforcing the need for integrated policies focused on early diagnosis, territorial surveillance, and longitudinal care.

The methodological diversity identified in the analyzed studies is substantial, encompassing interviews (32), parasitological analyses (33), morphophysiological assessments (34), geospatial analyses (35), and innovative approaches such as the ArtScience workshop, which integrates art and science in knowledge production (36). Despite this plurality, a structural limitation persists: a large proportion of the research remains confined to one or two One Health components, failing to simultaneously integrate the human, animal, and environmental dimensions.

This disconnect highlights an inconsistency between the use of the term *One Health* as a conceptual descriptor and its effective operationalization in scientific practice. Although widely disseminated, the concept continues to be applied in a partial and limited manner, as noted by Destoumieux-Garzón et al. (37). There is a clear predominance of studies focused on biomedical or veterinary dimensions, to the detriment of more comprehensive analyses that incorporate sociocultural, behavioral, political, and economic factors, components that are fundamental to the full implementation of the One Health approach (38). In this regard, the proposal of *Structural One Health* (39) broadens the analytical scope by integrating the historical, economic, and political determinants of emerging diseases, thereby revealing persistent methodological and epistemological gaps in the literature. This diagnosis reinforces the need to invest in interdisciplinary training, through the incorporation of One Health principles into undergraduate curricula, not only in health-related fields but also in agricultural, environmental, and social sciences programs.

Although scientific production on Chagas disease has achieved significant technical advances, a sectoral and fragmented logic still predominates. Research on epidemiological surveillance, entomology, human and canine serology, vector genotyping, molecular diagnostics, and climate modeling demonstrates considerable methodological sophistication, yet often lacks integration across the human, animal, and environmental components. This fragmentation constrains the ability to capture the full complexity of transmission processes and to inform interventions that are truly integrated, sustainable, and cost-effective.

This scenario calls for a critical reflection on the limits of compartmentalized science in addressing contemporary health challenges. Chagas disease is embedded in contexts marked by social inequality, ecological vulnerability, and fragile health systems, rendering reductionist approaches insufficient. The effective adoption of the One Health approach offers a promising pathway by integrating human, animal, and environmental dimensions, enabling the identification of shared risks, co-benefits, trade-offs, and concrete opportunities for equitable and transformative strategies (1).

## 5 Limitations

This review presents some limitations that should be considered when interpreting the findings. The exclusion of gray literature may have introduced publication bias, as studies with non-significant or negative findings are less likely to be published, although the search across multiple databases enhanced the comprehensiveness of study identification. Publication bias was not formally assessed, as the absence of quantitative synthesis precluded the use of standard evaluation methods.

Data extraction was performed by a single reviewer, which may have increased the risk of errors or subjective bias, although standardized procedures were applied to minimize inconsistencies.

A formal assessment of methodological quality or risk of bias was not performed due to the substantial methodological heterogeneity of the included studies, which limited the applicability of standardized assessment tools. We acknowledge that this represents a deviation from the initially registered protocol. Furthermore, the certainty of evidence was not evaluated using the GRADE approach, given the descriptive and integrative nature of this review.

### 5.1 Qualitative bias considerations

Based on a subjective appraisal, the included studies were considered to have moderate methodological quality. Entomological and molecular studies demonstrated greater rigor, with well-defined methods and consistent findings regarding vectors, reservoirs, and transmission dynamics. In contrast, qualitative and participatory studies, although essential to the One Health approach, showed limitations in methodological reporting and bias control. Epidemiological studies, in turn, exhibited weaknesses in the control of confounding factors and in sample representativeness, thereby limiting causal inferences.

The main sources of bias identified were selection bias, information bias, and limited comparability across studies. Nevertheless, the convergence of findings (particularly regarding the relationship between socioenvironmental vulnerability, vector presence, and the role of animals in transmission) supports the consistency of the evidence. Although the results should be interpreted with caution, the analysis indicates that the body of evidence supports the conclusions of this review, while also highlighting the need for greater methodological rigor in future One Health research.

## 6 Conclusion

The analysis of scientific production on Chagas disease from a One Health perspective reveals important advances while also highlighting persistent structural gaps in the integrated management of this complex condition. Although sectoral approaches have generated relevant technical knowledge, the predominance of fragmented frameworks continues to limit the effectiveness of surveillance, prevention, and treatment strategies. In contexts marked by intersecting social, ecological, and health vulnerabilities, addressing Chagas disease requires an approach that fully recognizes and operationalizes the interdependence among human, animal, and environmental systems.

This study highlights the urgency of consolidating One Health as a central pillar of public policies and research agendas addressing neglected tropical diseases, particularly Chagas disease. Beyond its conceptual value, the One Health approach holds strategic potential to enhance the efficiency, coordination, and sustainability of public health systems by strengthening integrated surveillance, promoting prevention-oriented care, and fostering intersectoral governance.

The reflections presented in this study seek to support the development of a more integrated and socially committed scientific practice, capable of addressing health inequities and tackling the structural determinants of neglected diseases. Strengthening One Health is not only a scientific imperative but also a political and ethical commitment to more equitable, sustainable, and transformative health interventions, aligned with the health and socioenvironmental challenges of the twenty-first century.

## Data Availability

The original contributions presented in the study are included in the article/Supplementary Material, further inquiries can be directed to the corresponding authors.
