# Supplementary material for: One health approach to chagas disease: a systematic review on the integration of human, animal, and environmental health
Source: Front Health Serv. 2026 Jun 16;6:1818840. doi: 10.3389/frhs.2026.1818840 (PMC13315012; doi:10.3389/frhs.2026.1818840)
Supplement: Supplementary file 1 [file Table1.docx]

**Supplementary Table 1.** Characterization of the scientific studies selected for the systematic review on Chagas disease with a focus on the One Health approach.

| **No.** | **Article Title** | **Year** | **Primary Author(s)** | **Study Type** | **Study Location** | **Method Used** | **One Health Components Addressed** | **Notes** | **Database** | | | |
| --- | --- | --- | --- | --- | --- | --- | --- | --- | --- | --- | --- | --- |
|  |  |  |  |  |  |  |  |  | **Web of Science®** | **Pubmed®** | **Dimensions®** | **Scopus®** |
| 1 | Living labs for migrant health research: the challenge of cocreating research with migrant population and policy makers | 2024 | Giménez, L.; Evangelidou, S.; Gresle, AS; de la Torre, L.; Ubalde-López, M.; Recasens, O.; Muñoz, E.; Pinazo, MJ; Requena-Méndez, A. | Original - Qualitative field study | Various locations (including immigrant communities) | Living labs, interviews, workshops with migrants and decision-makers | Human | Participatory approach, resonates with social and human One Health, but without a specific focus on vectors. | X |  |  |  |
| 2 | Socio-environmental factors associated with occurrence of triatomines in an endemic municipality, northern Minas Gerais, Brazil | 2024 | Gonçalves, TD; Ursine, RL; Matos, RLFD; et al. | Original - Epidemiological and environmental study | Minas Gerais, Brazil | Entomological survey, analysis of socio-environmental variables | Environmental + Animal + Human | Correlation between socioeconomic conditions and the presence of vectors; reinforces OH integration | X |  |  |  |
| 3 | One Health Approach to Toxoplasmosis: Owner and Dog Seropositivity as Spatial Indicators of Risk Areas | 2024 | Sohn-Hausner, N.; Correa, RG; Kmetiuk, L.B.; et al. | Original - Field and serological study | Brazil (Sao Paulo) | Serology in dogs and owners, georeferencing of occurrence | Environmental + Animal + Human | OH application in toxoplasmosis, highlights human and animal health synergy with spatial indicators | X | X |  |  |
| 4 | Incorporating an intersectional gender approach to improve access to maternal and child health screening services | 2024 | Arrivillaga, MR; Gold, M.; Rivera, EP; et al. | Field study | Latin America | Participatory methodology / Evaluation of policies with a gender approach in maternal and child health | Human | Interrelationship of various social, cultural and environmental determinants that influence the provision of health services, as well as the dynamics between traditional and institutional health systems. | X |  |  |  |
| 5 | Hospital case fatality and mortality related to Chagas disease in Brazil over two decades | 2024 | de Souza, EA; da Cruz, MM; Ferreira, AF; et al. | Original - Epidemiological study | Brazil | Retrospective analysis of hospital data | Human | High lethality observed, with regional and socioeconomic differences. | X |  |  |  |
| 6 | Presence of *T. cruzi TcI* and *T. dionisii* in wild bats from Yucatán, Mexico | 2024 | Moo-Millan JI et al. | Original - Field study | Mexico | PCR and molecular characterization in bats | Animal | Contributes to the ecological understanding of wild transmission. |  | X |  |  |
| 7 | One Health Approach to Toxoplasmosis (dogs and owners) | 2024 | Sohn-Hausner N et al. | Original - Field study | Latin America | Comparative serology between dogs and owners | Animal + Human | Clear study from the OH perspective on zoonotic infection. |  |  | X |  |
| 8 | Entomological indicators and food sources of triatomines in semi-arid Brazilian | 2024 | Silva LRSD et al. | Original - Entomological study | Brazil | Insect collection and food analysis | Animal | Relevant study in an endemic and vulnerable area. |  | X |  |  |
| 9 | Zoonotic infections at animal-human interface of primate trafficking in Peru | 2024 | Mendoza AP et al. | Original - Field study | Peru | Primate sampling and diagnosis | Animal | Emblematic case of the trafficking-zoonosis-OH interface. Not directly linked to CD. |  | X |  |  |
| 10 | Socio-environmental factors associated with triatomines in Minas Gerais | 2024 | Gonçalves TDS et al. | Original - Epidemiological study | Brazil | Multivariate analysis of socio-environmental factors | Environmental + Human | Highlights social and environmental factors in the presence of vectors. |  | X |  |  |
| 11 | New Triatomaspecies identified in Belize | 2024 | Gunter SM et al. | Original - Taxonomic study | Belize | Morphology + molecular genetics | Animal | Contribution to systematics and vector surveillance. |  | X |  |  |
| 12 | Addressing Chagas disease from a One Health perspective: risk factors, lessons learned and prevention of oral transmission outbreaks in Colombia | 2024 | Olivera, Mario J. et al. | Mixed study: ecological epidemiological analysis and semi-structured interview |  | Scientific review combined with analysis of documented oral outbreaks in the country | Environmental + Animal + Human | The article highlights the need for integrated actions to prevent oral transmission of T. cruzi, analyzing past outbreaks from the perspective of ecological, behavioral, and health factors. It reinforces the practical application of the One Health concept. |  |  | X | X |
| 13 | Occurrence of triatomines in public spaces: An atypical case in the Southwestern Brazilian Amazon | 2023 | da Cruz, KS; Ribeiro, MAL; Madeira, FP et al. | Original - Field study | Brazilian Amazon | Collection of triatomines in squares, schools, and public areas | Animal | Reports the presence of vectors in urban spaces, highlighting the risk to public health due to the environment-community interface. | X |  |  |  |
| 14 | Social determinants of Chagas disease in Costa Rica | 2023 | Solano, AMR; García, HAC | Secondary data analysis | Costa Rica | National data | Environmental + Human | Highlights inequalities, access to health and social determinants. | X |  |  |  |
| 15 | Stage-dependent feeding behavior in a wild vector of Chagas disease | 2023 | De Bona S et al. | Original - Field study (original) | South America | Feeding behavior analysis of triatomines | Animal | Explores vector behavior with ecological implications. |  | X |  |  |
| 16 | Vector mapping and bloodmeal metabarcoding in Caracas | 2023 | Segovia M et al. | Original - Field study | Venezuela | Blood metabarcoding and geographic mapping | Environmental + animal | Addresses the complexity of vector transmission in an urban context. |  | X |  |  |
| 17 | *T. cruzi* infection in mammals in Florida | 2023 | Torhorst CW et al. | Field study (original) | USA (Florida) | PCR in wild animals | Animal | Expands knowledge about wildlife cycles in the southeastern US. |  | X |  |  |
| 18 | Severe Chagas disease in Ecuador: a countrywide geodemographic analysis | 2023 | Vásconez-González J et al. | Epidemiological study (original) | Ecuador | Geospatial analysis of clinical data | Human | Maps the distribution and severity of chronic cases in the country. |  | X |  |  |
| 19 | Chirimacha Alert: triatomine surveillance system | 2023 | Tamayo LD et al. | Applied study (information system) | Peru | Surveillance app development | Animal + Human | Participatory technology for urban vector control. |  | X |  |  |
| 20 | Humans as blood-feeding sources in sylvatic triatomines in Chile | 2023 | San Juan E et al. | Entomological study (original) | Chile | Blood feeding analysis | Animal | Demonstrates human contact with wild vectors — risk of transmission. |  | X |  |  |
| 21 | Diet of sylvatic triatomine Mepraia spinolainear human settlements | 2023 | Sierra-Rosales C et al. | Entomological study (original) | Chile | Intestinal content analysis | Animal | Reflects risks of rural-human interface with wild vectors. |  | X |  |  |
| 22 | Perceptions of problems with household insects in Peru | 2023 | Castillo-Neyra R et al. | Qualitative study (original) | Peru | Interviews and participatory research | Human | Assesses community perception — valuable for health education initiatives. |  | X |  |  |
| 23 | Xenotoxication in dogs to block T. cruzi (preprint and published version) | 2023 | Rokhsar JL et al. | Experimental study (original) | Argentina | Fluralaner trials in dogs | Animal | Innovative strategy with positive side effect in vector control. |  | X |  |  |
| 24 | A multidisciplinary, collaborative, inter-agency and comprehensive approach for the control of Chagas Disease as a public health problem in Guatemala | 2022 | Monroy, MC; Penados, D.; Pineda, J.; et al. | Intervention study/program (original) | Guatemala | Multisectoral intervention, partnerships, indicators | Environmental + Animal + Human | National coordination model for sustained Chagas disease control. | X |  |  |  |
| 25 | Incentivizing optimal risk map use for Triatoma infestans surveillance in urban environments | 2022 | Arevalo-Nieto, C.; Sheen, J.; Condori-Luna, GF; et al. | Urban surveillance and planning study (original) | Bolivia | Development and use of risk maps for surveillance | Environmental | Risk map as an operational tool for participatory surveillance OH | X | X |  |  |
| 26 | Trypanosomatid parasites in mammals in Colombia | 2022 | Castillo-Castañeda AC et al. | Field study (original) | Colombia | PCR and identification of parasites in mammals | Animal + Human | Maps the diversity of trypanosomatids in wildlife. |  | X |  |  |
| 27 | Spatial distribution of triatomines in urban area of Montes Claros | 2022 | Campos MCOA et al. | Spatial study + laboratory (original) | Brazil | Georeferenced mapping | Environmental + Animal + Human | Shows urbanization of vector transmission — important for public policy. |  | X |  |  |
| 28 | Metagenomic investigation of ticks from Kenyan wildlife | 2022 | Ergunay K et al. | Metagenomic study (original) | Kenya | DNA sequencing in ticks | Environmental + animal | It does not directly focus on T. cruzi, but contributes to zoonotic understanding under OH. |  | X |  |  |
| 29 | Integrated approaches to howler monkey (Alouatta spp.) medicine in professional care and conservation | 2022 | -Jaramillo, Enrique et al. | Applied Modeling Study | Latin America | Discussion based on practical experiences and scientific literature on the management and conservation of primates at risk | Environmental + animal | This article addresses the medicine and conservation of howler monkeys under environmental stress caused by human activities. It offers an integrated approach to clinical care, conservation, and ecology, focusing on the connection between animal and environmental health. |  |  | X | X |
| 30 | Diversity of Trypanosoma cruzi parasites infecting Triatoma dimidiata in Central Veracruz, Mexico, and their One Health ecological interactions | 2021 | Murillo-Solano, C.; Ramos-Ligonio, A.; López-Monteon, A.; Guzmán-Gómez, D.; Torres-Montero, J.; Herrera, C.; Dumonteil, E. | Field and molecular study (original) | Veracruz, Mexico | Metabarcoding, deep sequencing: identification of T. cruzi strains, blood sources, and vector microbiota | Animal | It demonstrated feeding dynamics with multiple hosts and several DTUs, integrating environmental, animal and human components. | X | X |  |  |
| 31 | Chagas Express XXI: a new ArtScience social technology for health and science education | 2021 | Araujo-Jorge, TC; Ferreira, RR; Rocha, RCM; et al. | Participatory educational intervention study (original) | Brazil | ArtScience activities, community education and active pursuit | Environmental + Animal + Human | Innovative education and community engagement tool for OH case detection | X |  |  |  |
| 32 | The connection between Trypanosoma cruzi transmission cycles by Triatoma brasilensis brasilensis... | 2021 | Lima-Neiva, V.; Toma, HK; Aguiar, LMA; et al. | Field study (original) | Brazil | Vector study, ecological habitat analysis | Environmental + animal | It shows an emerging risk in an area with adverse climate variations for biodiversity. | X |  |  |  |
| 33 | Use of citizen science in vector surveillance: experience with triatomines in Mexico | 2021 | Salazar-Schettino, PM; Bucio-Torres, MI; et al. | Social innovation study | Mexico | Collaborative data platform | Animal + Human | Community engagement via citizen science shows potential for participatory surveillance. | X |  |  |  |
| 34 | First molecular detection of *T. cruzi* , *T. rangeli* and *Leishmania spp.* in capybaras | 2021 | Ferrer E et al. | Field study (original) | Brazil | PCR in capybara samples | Animal | It highlights the role of capybaras as reservoirs and the presence of multiple zoonotic parasites. |  | X |  |  |
| 35 | Risk of T. cruziinfection among travelers visiting Latin America | 2021 | Sánchez-Montalvá A et al. | Cross-sectional study (original) | Europe | Review + clinical cases | Human | Traveler risk study with implications for international surveillance. |  | X |  |  |
| 36 | Vector-borne pathogens among US Government Working Dogs | 2021 | Meyers AC et al. | Serological study (original) | USA | Serology in operational dogs | Animal | Alert for dog exposure and possible spillovers. |  | X |  |  |
| 37 | Preliminary Characterization of Triatomine Bug Blood Meals on the Island of Trinidad | 2020 | Hylton, A.; Fitzpatrick, DM; Suepaul, R.; et al. | Laboratory study (original) | Trinidad - Caribbean | Analysis of ingested blood by PCR and sequencing | Animal | Opportunistic feeding behavior connects vectors to multiple hosts. | X |  |  |  |
| 38 | Biogeographic distribution of Chagas disease vectors in South America under climate change scenarios | 2020 | Ceccarelli, S.; Rabinovich, J.E. | Ecological modeling (original) | South America | Species distribution models with climate scenarios | Environmental | Anticipation of changes in vector distribution under climate change, useful for OH planning | X |  |  |  |
| 39 | Economic burden of Chagas disease in endemic countries | 2020 | Lee, BY; Bacon, KM; Bottazzi, ME; Hotez, P.J. | Economic evaluation (original) | Latin America | Direct and indirect cost modeling | Human | It highlights the high costs of not treating/preventing the disease — a strong argument for OH. | X |  |  |  |
| 40 | Predicting distribution of triatomine vectors under climate change scenarios | 2020 | Ceccarelli, S.; Medone, P.; et al. | Ecological modeling | South America | Ecological niche modeling with climatic variables | Environmental + animal | Climate change could expand risk areas — a critical issue in OH. | X |  |  |  |
| 41 | Dispersal patterns of T. cruzi in Arequipa, Peru | 2020 | Berry ASF et al. | Genetic-spatial study (original) | Peru | Phylogeographic and spatial analysis | Animal | Addresses the spatiotemporal dynamics of urban transmission. |  | X |  |  |
| 42 | Mechanism of T. cruzi acquisition by triatomines | 2020 | Tustin AW et al. | Experimental study (original) | Peru | Controlled infection experiment | Environmental | Studies the biology of vector transmission in detail. |  | X |  |  |
| 43 | Triatoma costalimai Naturally Infected by Trypanosoma cruzi: A Public Health Concern | 2019 | Teves, SC; Toma, HK; Lopes, C.; de Oliveira, BLN; et al. | Field study (original) | Brazil | Bioassay, natural infection, microscopic/molecular identification | Animal | Highlights infected wild/domestic vector, sign of urban risk. | X |  |  |  |
| 44 | Identification of Triatomines and Their Habitats in a Highly Developed Urban Environment | 2019 | Dye-Braumuller, K.C.; Gorchakov, R.; Gunter, SM; et al. | Entomological field study (original) | USA (urban environment) | Trapping and mapping + molecular analysis | Animal | Vectors detected in highly urban environments, reinforcing OH surveillance. | X |  |  |  |
| 45 | Trypanosoma cruzi in synanthropic mammals of urban environments | 2019 | Xavier, SCC; Roque, ALR; et al. | Ecological study | Brazil | Capture and testing of synanthropic mammals | Animal | Highlights new hosts in urban areas — warning of silent risk. | X |  |  |  |
| 46 | Giardia Infection and Trypanosoma cruzi Exposure in Dogs in Nicaragua | 2019 | Roegner AF et al. | Observational study (original) | Nicaragua | Serology and parasitology in dogs | Animal | Demonstrates zoonotic coinfection with public health importance. |  | X |  |  |
| 47 | Polyparasitism and zoonotic parasites in dogs in the Argentine Chaco | 2019 | Enriquez GF et al. | Field study (original) | Argentina | Coproparasitological diagnosis | Animal | High zoonotic parasite load in vulnerable area. |  | X |  |  |
| 48 | Occurrences of triatomines and first reports of Panstrongylus geniculatus in urban environments in the city of São Paulo, Brazil | 2018 | Ceretti, W.; Vendrami, DP; de Matos, MO; et al. | Field study (original) | Sao Paulo, Brazil | Entomological collection in urban areas, occurrence report | Animal | Pioneering record of P. geniculatus in an urban context, emerging risk | X |  |  |  |
| 49 | Continuing evidence of Chagas disease along the Texas-Mexico border | 2018 | Nolan, MS; Aguilar, D.; Brown, E.L.; et al. | Original study | Texas/Mexico | Human serology, vector study, epidemiological analysis | Human | Confirms endemic pattern and need for continuous interdisciplinary surveillance. | X |  |  |  |
| 50 | Access to diagnosis and treatment of Chagas disease in non-endemic countries: the case of Switzerland | 2018 | Jackson, Y.; Angheben, A.; Carrilero, Fernández B.; et al. | Health Access Study (original) | Switzerland | Interviews, health system analysis | Human | Focuses on challenges faced by migrants in accessing health care – an emerging issue in OH. | X |  |  |  |
| 51 | Interdisciplinary network engagement in a non-formal education process to study foodborne Chagas disease puzzle in Brazilian Amazon | 2018 | Araújo-Jorge, Tânia Cremonini de et al. | Interdisciplinary qualitative study | Brazil – Amazon | Experience report and analysis of an interdisciplinary educational network | Animal + Human | Study with emphasis on community participation and integration of knowledge from the One Health perspective. |  |  | X |  |
| 52 | One Health Interactions of Chagas Disease Vectors, Canid Hosts, and Human Residents along the Texas-Mexico Border | 2016 | Garcia, MN; O'Day, S.; Fisher-Hoch, S.; et al. | Field study (original) | Texas/Mexico | Vector collection, serology in dogs and humans | Animal | Shows zoonosis in transboundary interface; dogs as sentinels. | X | X |  |  |
| 53 | A cellular automaton model for the transmission of Chagas disease in heterogeneous landscape... | 2016 | Cissé, B.; El Yacoubi, S.; Gourbiere, S. | Mathematical modeling study | Theoretical, applicable to diverse landscapes | Cellular automaton modeling with ecological parameters | Environment + Animal | Allows for the simulation of vector and zoonotic dynamics under different scenarios, OH agenda for policies. | X |  |  |  |
| 54 | Epidemiology of and Impact of Insecticide Spraying on Chagas Disease in Communities in the Bolivian Chaco | 2013 | Samuels, AM; Clark, E. H.; Galdos-Cardenas, G.; et al. | Cross-sectional study (original) | Bolivia | *T. cruzi* infection and examine age-specific prevalence to estimate the strength of infection | Environmental + Human | As in other areas of the Chaco, we found an extremely high prevalence of Chagas disease. | X |  |  |  |
| 55 | Evaluating risk factors for household infestation by triatomines in Brazil | 2016 | Abad-Franch, F.; Santos, WS; et al. | Epidemiological study | Brazil | Multivariate modeling and household data | Environmental + animal | Structural factors of houses and the presence of animals correlate with infestation. | X |  |  |  |
| 56 | Habitat Management to Reduce Human Exposure to T. cruzi | 2016 | Shender L et al. | Environmental intervention study | Bolivia | Environmental modifications and entomological assessment | Environmental + animal | Demonstrates risk reduction through environmental management in rural communities. |  | X |  |  |
| 57 | Molecular Diversity of T. cruzi in Triatoma protracta (California) | 2016 | Shender LA et al. | Molecular study | USA (California) | PCR and sequencing | Animal | Evidence of genetic diversity in wild vector in the USA. |  | X |  |  |
| 58 | Integrating an infectious disease program into the primary health care service: a retrospective analysis of Chagas disease community-based surveillance in Honduras | 2015 | Hashimoto, K.; Zúniga, C.; Nakamura, J.; Hanada, K. | Original study | Honduras | Analysis of primary surveillance data, health indicators | Human | Assesses the integration of Chagas into the primary care system. | X |  |  |  |
| 59 | Role of poultry in peridomestic transmission of Trypanosoma cruzi | 2015 | Gürtler, RE; Kitron, U.; et al. | Ecological study | Argentina | Monitoring in environments with birds | Animal | Birds as an ecological barrier (not hosts), but attract vectors — a complicating factor. | X |  |  |  |
| 60 | Community-based surveillance systems for Chagas disease in Guatemala | 2014 | Hashimoto, K.; Yoshioka, K.; et al. | Program evaluation | Guatemala | Community system implementation and analysis | Environmental + Human | Active community participation improves efficiency and early response. | X |  |  |  |
| 61 | Population structure of the Chagas disease vector, Triatoma infestans, at the urban-rural interface | 2013 | Foley, EA; Khatchikian, CE; Hwang, J.; Ancca-Juárez, J.; et al. | Population/genetic study | Peru (urban-rural interface) | Genotyping of vector populations; population analysis | Animal | Reveals exchange between vector populations, important for adapted control. | X |  |  |  |
| 62 | House infestation and natural infection by Triatoma brasiliensis in Northeast Brazil | 2013 | Monteiro, FA; Weirauch, C.; Felix, M.; et al. | Entomological study | Northeast Brazil | Home collection, analysis of natural infection | Environmental + Animal + Human | It shows direct risk to vulnerable populations in precarious housing | X |  |  |  |
| 63 | Domestic dogs as sentinels for Trypanosoma cruzi transmission | 2013 | Estrada-Franco, JG; Garg, N.; et al. | Surveillance study | Mexico | Serological tests in dogs | Animal | Dogs as useful bioindicators in environmental and epidemiological surveillance. | X |  |  |  |
| 64 | Coatis as potential reservoir of T. cruziin Costa Rica | 2013 | Mehrkens LR et al. | Original study | Costa Rica | PCR in wild coatis | Animal | Evidence of the ecological role of wild mammals in transmission. |  | X |  |  |
| 65 | Global Trends in the Use of Insecticides to Control Vector-Borne Diseases | 2012 | van den Berg, H.; Zaim, M.; Yadav, RS; et al. | Secondary data | World | Analysis of historical use and regulations of insecticides | Human + Environmental + Animal | Addresses environmental and human impacts of chemical controls; relevance to OH policy. | X |  |  |  |
| 66 | American trypanosomiasis and associated risk factors in dogs owned from the major city of Yucatan, Mexico | 2015 | Jiménez-Coello, M.; Guzmán-Marin, E.; Ortega-Pacheco, A.; et al. | Serological study | Mexico (Yucatan) | Serological tests in dogs | Animal | Dogs as sentinels of infection; risk to rural human population | X |  |  |  |
| 67 | Integration of vector surveillance and control into primary health care services | 2011 | Hashimoto, K.; Schofield, C.J. | CAP Study | Latin America (regional experiences) | Analysis of public health strategies | Environmental + Human | Integrating health systems requires health professionals to go beyond their usual responsibilities and acquire management skills. | X |  |  |  |
| 68 | Spatio-temporal variability of NDVI-precipitation over southernmost South America: possible linkages between climate signals and epidemics | 2008 | Tourre, YM; Jarlan, L.; Lacaux, JP; et al. | Ecological/climatic study | South America (South) | Time series analysis of NDVI and precipitation; epidemic modeling | Environmental | Relates environmental patterns to possible outbreaks, provides support for vector forecasting under OH | X |  |  |  |

**Supplementary Box 1.** Examples of Study Categorization

| **Study reference** | **Category** | **Reasons for Categorization** |
| --- | --- | --- |
| Gonçalves, T. D. S., Ursine, R. L., Cardozo, M., Matos, R. L. F. D. R., de Souza, R. D. C. M., Diotaiuti, L. G., ... & Vieira, T. M. (2024). Socio‐environmental factors associated with the occurrence of triatomines (Hemiptera: Reduviidae) in an endemic municipality in northern Minas Gerais, Brazil. *Zoonoses and Public Health*, 71(1), 34-47. doi: <https://doi.org/10.1111/zph.13081> | Animal, Human, and Environmental Health | The animal dimension was addressed through entomological data derived from active and passive surveillance of triatomines. The environmental dimension was incorporated through the analysis of changes in land use and land cover, aiming to assess whether these changes may influence the presence of triatomines. The human dimension was integrated through sociodemographic indicators related to population exposure and vulnerability (such as household economic income, number of domiciles, and the presence of pavement, electricity, and street lighting). The classification considered the articulation among these dimensions, as evidenced by the analysis of the relationship between socioeconomic conditions, vector presence, and land cover conditions. |
| Sohn-Hausner, N., Correa, R. G., Kmetiuk, L. B., da Silva, E. C., de Moraes, G. N., Rocha, G. D. S., ... & Biondo, A. W. (2024). One health approach to toxoplasmosis: Owner and dog seropositivity as spatial indicators of risk areas for acquired, gestational and congenital transmission. *Tropical Medicine and Infectious Disease*, 9(7), 143. doi: <https://doi.org/10.3390/tropicalmed9070143> | Animal, Human, and Environmental Health | The animal dimension was represented by seropositivity in dogs. The human dimension was represented by seropositivity in their owners. The environmental dimension was addressed through the use of spatial analysis and the identification of risk factors in the domestic environment. Analytical integration was evidenced by the use of dogs as epidemiological sentinels, directly linking animal findings to the occurrence of human cases in specific environmental contexts, thereby characterizing interdependence among the three dimensions. |
| Arrivillaga, M. R., Gold, M., Rivera, E. P., & Juárez, J. G. (2024). Incorporating an intersectional gender approach to improve access to maternal and child health screening services. *International journal for equity in health*, 23(1), 32. doi: <https://doi.org/10.1186/s12939-024-02109-3> | Human Health | Although this study addresses social, cultural, and institutional determinants through a participatory methodology, there is no analytical incorporation of data, indicators, or results related to animal or environmental health. Therefore, according to the adopted operational rule, the absence of integration among dimensions justified its classification as unidimensional. |
